# Motor Imagery Acquisition and Classification Using a Low-Cost 8-Channel EEG System in a VR ADHD Serious Game Environment: A Case Study

**DOI:** 10.3390/s26175576

**Published:** 2026-09-02

**Authors:** Lukas Röhrling, Selina Breuer, Carina Arnberger, Christoph Aigner, Thomas Grechenig, René Baranyi

**Affiliations:** 1 Research Group for Industrial Software (INSO), TU Wien, Wiedner Hauptstrasse 76/2/2, 1040 Vienna, Austria; lukas.roehrling@rise-world.com (L.R.); selina.breuer@inso-world.com (S.B.); carina.arnberger@inso-world.com (C.A.); christoph.aigner@inso-world.com (C.A.); thomas.grechenig@inso-world.com (T.G.); 2 Research Industrial Systems Engineering (RISE), Concorde Business Park F, 2320 Schwechat, Austria; 3 RISE Institute of Technology (RIT), Sanskrithi School of Engineering (SSE), Puttaparthi 515134, India

**Keywords:** Brain–computer-Interface, motor imagery, electroencephalography, machine learning

## Abstract

Attention-deficit/hyperactivity disorder (ADHD) involves difficulties in sustaining attention and resisting distraction. This has motivated the development of feedback-driven environments for cognitive control training. Integrating electroencephalography (EEG) sensors into Virtual Reality (VR) serious games for cognitive therapy remains relatively underexplored and requires reliable, non-invasive brain–computer interfaces. The existing solutions use multi-channel systems that primarily suffer from requiring complex hardware, while not combining motor imagery (MI) with concentration levels. Therefore, this case study evaluates the feasibility and data quality of a lightweight, cost-effective sensor configuration for real-time control of mental state. A non-invasive, eight-channel OpenBCI Cyton board was integrated with an EEG cap using the international 10–20 placement system, alongside a Meta Quest 2 headset, to capture MI and concentration signals directly from the user’s scalp. Signal acquisition was hindered by high impedance and channel railing, which required conductive gel mitigation, while mechanical tension from the VR headset strap introduced motion artifacts and noise. Nevertheless, under stable signal conditions, the optimized eight-channel sensor setup achieved a subject-specific online classification accuracy of up to 90% using the deep learning model “EEGNet”. The findings demonstrate the technical feasibility of acquiring and classifying EEG activity using a low-cost eight-channel sensor configuration in an interactive VR-BCI Serious Gaming application, provided that skin–electrode impedance and mechanical sensor interferences are managed. The results provide a basis for future investigation of such systems in cognitive-training applications, while further studies, including clinical evaluations, are required to assess their applicability in therapeutic contexts.

## 1. Introduction

Mental and neurological disorders are becoming an increasing burden for affected individuals, healthcare institutions, and society in general [1,2]. According to estimates from the World Health Organization (WHO), mental conditions are expected to become the leading cause of global disease burden by 2030 [1]. One relevant neuro-developmental disorder in this context is “Attention Deficit Hyperactivity Disorder (ADHD)”, which affects approximately 5.8% to 10% of children worldwide and accounts for nearly 30% to 40% of all referrals to outpatient child and adolescent psychiatric clinics [3,4]. It is characterized primarily by persistent symptoms such as hyperactivity, inattention, and impulsivity that are not appropriate for the child’s developmental stage [5]. In untreated cases, ADHD symptoms persist into adulthood in around 60% to 65% of patients [6].

Given these challenges, Brain–Computer Interfaces (BCIs) have become an interesting approach to digital interaction and clinical rehabilitation [7,8]. Originally, BCIs were mainly developed for people with severe motor impairments, such as Amyotrophic Lateral Sclerosis (ALS) or stroke-induced paralysis, to support communication and interaction [8]. In recent years, their use has also expanded toward neurofeedback systems [9]. For ADHD, brain activity can be integrated into serious games, where neural signals are used to provide real-time feedback to users and therapists [10,11]. This can support the training of attention and self-regulation in a more interactive and motivating way [9]. Serious games are digital games that contain more than just entertainment aspects by pursuing additional objectives and goals, such as learning, training, rehabilitation, or behavior change [12]. In digital health, they can promote and support various aspects, such as physical activity, medical education and simulation, lifestyle behaviors, and diagnostic learning [13]. Within rehabilitation, serious games can make therapy more engaging and interactive, often incorporating gamified elements that help support motivation and adherence [14]. Based on continuous electroencephalography (EEG) signal acquisition, these specially developed applications can adapt to the participant’s cognitive state, for example, by automatically pausing or resuming the gameplay when the estimated attention level falls below the predefined threshold [10,11].

Recent developments in consumer electronics and sensor technologies make it easier to build mobile and interactive neurotechnology systems [15,16]. The combination of standalone Virtual Reality (VR) head-mounted displays (HMDs) and mobile EEG hardware enables the creation of fully immersive, closed-loop serious games [17]. These systems can capture real-time neurophysiological data, process cognitive metrics, and adapt the virtual environment to the user’s mental state [16]. This combination can create controlled therapeutic environments that reduce external visual distractions and help users stay engaged during training [17,18]. This is especially relevant for individuals who struggle with sustained attention, since external distractions can interfere with therapeutic exercises. By reducing such distractions, these environments can provide more controlled conditions for attention-focused training [18].

To enable the control of such applications solely through cognitive activity, non-invasive BCIs acquire electrical brain signals directly from the patient’s scalp using EEG electrodes [19]. EEG signals reflect electrical activity generated by neural communication processes in the brain, which are essential for cognitive, sensory, and motor functions [20]. Moving the left foot, closing the eye, or just the using one’s imagination to raise one’s arms, representing motor imagery (MI), leads to measurable changes in electrical activity within specific cortical regions. These signals are primarily generated in the brain through the synchronized activity of large populations of cortical neurons and can be recorded directly from the scalp by measuring the electrical potential differences associated with neural activity [21]. EEG signals are highly susceptible to physiological and environmental noise; as a result, factors such as electrode movement, eye blinks, muscle activity, power-line interference, and nearby electronic devices can significantly affect the quality of the measured brain activity [20]. Consequently, the recorded EEG data must undergo several processing steps, and the resulting predictions can be translated into digital control commands, enabling users to interact intuitively with digital systems without requiring muscular effort, using only their minds [8].

Despite these promising possibilities, several critical challenges restrict the widespread accessibility and adoption of BCI systems in daily therapeutic plans. Traditional medical-grade systems are limited by high costs and restricted availability, confining them primarily to highly specialized clinical or laboratory settings [19]. Furthermore, the market remains sharply divided between invasive devices, which require surgical implantation and present inherent medical risks, and non-invasive devices, which are safe but suffer from lower signal quality, primarily due to noise and environmental artifacts [22]. Beyond cost and accessibility, the practical usability of non-invasive setups is heavily compromised by institutional complexity [16]. Commonly used BCI setups typically rely on dense electrode arrays following standardized EEG placement systems, which require lengthy preparation times, precise physical adjustments, and constant technical monitoring, further reducing usability [19,23]. This complexity, paired with the discomfort of electrodes, primarily requiring wet conductive gel to establish sufficient impedance, poses a significant bottleneck for independent home use or rapid clinical integration, highlighting a pressing research gap for highly minimized, sparse-electrode configurations that balance user comfort with predictive accuracy [16,24,25].

Therefore, this work presents a case study of an adaptable, low-cost, 8-channel EEG-based Brain–Computer Interface system for acquiring and classifying MI tasks and estimating the user’s level of attention. Furthermore, the work investigates the key factors influencing reliable EEG signal acquisition and classification and discusses the practical challenges associated with deploying low-cost BCI systems in interactive VR environments. The proposed approach is trained using data acquired from a single primary subject and is subsequently evaluated with five healthy participants using a VR Serious Game called “FormulaMind” [26].

### 1.1. Related Work

In recent years, the integration of VR into serious gaming has emerged as a powerful paradigm for cognitive rehabilitation and psychological therapy [27]. Serious games can benefit significantly from the unique affordances of immersive VR technology [28]. By utilizing HMDs, these applications isolate the user from the physical environment, effectively minimizing external visual and auditory distractions. This high degree of immersion creates a controlled, high-fidelity environment where cognitive performance can be systematically trained and monitored [27]. Previous studies have successfully deployed VR serious games to address various cognitive impairments. In the context of developmental disorders, immersive environments have proven highly effective in maintaining user engagement and motivation, both of which are critical to achieving long-term neuroplastic adaptation and therapeutic success [29,30].

Building upon these immersive environments, BCIs have fundamentally expanded the capabilities of neurofeedback, establishing a direct communication pathway between cortical activity and digital applications [16]. Traditional neurofeedback therapies for ADHD primarily focus on modulating specific EEG frequency bands, such as reducing the theta-to-beta ratio (θ/β) over the frontal cortex to improve sustained attention and reduce impulsivity [10]. Researchers have increasingly integrated EEG-based neurofeedback into interactive serious games, allowing users to control virtual environments through measured brain activity. Examples include racing games, attention-based navigation tasks, and rehabilitation-oriented serious games that adapt gameplay based on measured EEG activity [31,32]. Furthermore, MI-based applications have shown promise in supporting rehabilitation and assisting individuals with motor impairments by translating classified EEG patterns into meaningful control commands [21,33]. But current systems often provide only a limited representation of the user’s cognitive state, leaving a clear research gap regarding multimodal neurofeedback frameworks capable of simultaneously processing MI and concentration information within an immersive VR environment [31,32].

From a technical perspective, non-invasive EEG remains the dominant signal acquisition method for BCI-controlled serious games because it enables real-time measurement of cortical activity while maintaining comparatively low hardware costs and good portability. Nevertheless, EEG-based systems remain susceptible to artifacts, individual variability, and reduced spatial resolution, making robust signal processing and classification essential for reliable interaction [15,21]. Recent studies have demonstrated that practical BCI applications can be implemented using consumer-grade EEG devices equipped with only a small number of electrodes, including various available systems, while still achieving promising classification performance in real-time serious game applications [31,32,33,34,35]. Therefore, the work presented here investigates whether an immersive VR serious game that combines MI and concentration-based neurofeedback can be implemented with an 8-channel EEG device, addressing the identified research gaps while maintaining a practical, cost-effective hardware configuration.

### 1.2. Novelty of Proposed Solution

The main contribution of this work is a case-study-based investigation of the deployment and optimization of a lightweight, highly cost-effective 8-channel EEG system within a BCI for the reliable acquisition and classification of MI and attention-related signals, tailored to an implemented serious game in an immersive VR environment. While state-of-the-art neurofeedback and MI systems typically rely on high-density, expensive clinical EEG equipment typically employing more than 10 to 19 channels, this case study demonstrates the feasibility of achieving a 2-class MI classification utilizing only 8 individual configurable electrodes by carefully focusing on their optimized placement, all defined according to the international 10–20 system [16] and in respect to the most relevant cortical regions for MI tasks. Powered by a highly mobile, low-cost hardware setup using the BCI technology of the OpenBCI Cyton board [36] and a Meta Quest 2 [37] HMD, the proposed framework offers an adaptable, stand-alone, and portable rehabilitation platform, enabling use beyond institutional laboratory settings.

Furthermore, this research also provides critical insights into the real-world boundaries and structural challenges of low-cost BCI integrations. We systematically address and analyze technical and environmental limitations within a VR and Serious Game Setting, which includes the aspects of sensor selection and signal integrity, performing a comparative analysis of dry versus wet electrode setups, highlighting the need for conductive gel to mitigate high skin–electrode impedance and prevent channel railing under the physical constraints and mechanical tension imposed by a VR headset strap. Furthermore, the influence of mechanical and environmental artifacts are highlighted, demonstrating the possible management of signal noise caused by loose electrode cables during head movements, as well as the identification of ambient electrical interference (e.g., from nearby laboratory or household appliances) that distorts the microvolt-range EEG signals. In addition, the transition from proprietary graphical user interfaces (OpenBCI GUI) to raw, code-driven data acquisition (via the BrainFlow SDK) and real-time algorithmic filtering optimized for low-latency standalone execution is analyzed.

A further contribution of this work is also the verification of the custom-built training pipeline. To train the predictive framework, a standardized data acquisition protocol was established, recording 900 subject-specific training trials per class/direction (left vs. right MI). By fitting and evaluating both shallow machine learning and advanced deep learning convolutional architectures (specifically, EEGNet [38]), this work demonstrates that a minimal 8-channel consumer-grade configuration can achieve an online classification accuracy of up to 90% using a personalized dataset. This bridges the gap between high-accuracy demands and practical user comfort or potential acceptance, which is considered important for modern interactive serious gaming and cognitive therapy.

## 2. Materials and Methods

The methodological framework of this case study builds upon a master’s thesis by Lukas Röhrling [26] and follows a multi-stage engineering and evaluation pipeline centered on the development of a sensor-driven serious game application. The overall process begins with the Hardware and Data Acquisition Setup, where the physical EEG sensor infrastructure is established. Following a structured Data Acquisition Protocol and Training Dataset phase, a corpus of 1800 trials was generated to train the system, which subsequently underwent rigorous Signal Processing and Feature Extraction to isolate relevant neural markers. These features were utilized for Deep Learning Model Fitting and Classification. These classified mental states and continuous concentration levels were then translated into real-time digital commands within a developed serious game. This technical sensor pipeline ultimately drives the Immersive VR Serious Game Interface, titled Formula Mind, which was evaluated across 36 experimental trials using both desktop PCs and VR platforms. This publication focuses on the sensor/signal and hardware aspects and does not cover details of the developed Serious Game, except for a general overview to help understand other aspects and the overall context.

### 2.1. Hardware and Data Acquisition Setup

EEG signals were captured using the OpenBCI Cyton board (OpenBCI, Brooklyn, NY, USA) [36], an 8-channel neural interface featuring a 32-bit processor and a programmable Bluetooth dongle for stable wireless data transmission, enabling the recorded signals to be transmitted to a computer. By default, the Cyton board acquires data at a sampling rate of 250Hz with a microvolt (µV) resolution.

To ensure reproducibility and standardize electrode placement, an elastic EEG cap manufactured by BrainBit (New York, NY, USA) [39] was utilized, incorporating the international 10–20 system [16]. Based on the spatial distribution of cortical sensorimotor rhythms, eight specific electrode channels were selected, F3,F4,C3,C4,Cz,Fz,O1, and O2, all covering the relevant cortical regions, which are necessary to detect the MI-relevant frequency bands (alpha, mu, and beta bands). Furthermore, earclip electrodes were attached to the lobes, serving as reference and ground pins to measure meaningful voltage differences. Due to high impedance between the electrodes and the skin, a comparative analysis between dry and wet electrode configurations was conducted. Therefore, despite first assumptions of using dry electrodes, the initial setup was optimized by applying a water-soluble conductive gel to the selected electrodes to achieve stable skin–electrode contact, reduce high impedance values below the critical threshold of 750kΩ, and prevent signal clipping (channel railing).

### 2.2. Data Acquisition Protocol and Training Dataset

A standardized calibration and recording protocol was implemented to establish a subject-specific training dataset. For the two-class MI task (imagining directional left vs. right hand/arm movements), a primary subject performed structured training sessions, applying the required training dataset to train classification models. The training dataset for the subsequent model training consists of

- A total of 1800 marked and recorded training trials, with 900 for left MI and 900 for right MI.
- Each trial represents continuous MI execution for 14 s.
- During offline data collection, the system continuously tracks signal quality. Training runs affected by severe motion artifacts, loose electrode cables, or environmental interference such as common ambient 50/60Hz power-line noise from laboratory appliances were automatically flagged via the code and discarded by a dedicated signal-quality check before being stored in the corresponding training dataset labeled as left or right MI.

### 2.3. Signal Processing and Feature Extraction

The raw EEG data stream recorded via electrodes connected to the OpenBCI Cyton board [36] is accessed directly via the open-source library BrainFlow SDK API [40] (v5.19) within a Python-Backend code, bypassing proprietary graphical user interfaces (OpenBCI GUI). The processing pipeline, implemented in Python, applies three different digital filtering stages, beginning with a bandpass filter ranging from 8Hz and 30Hz to retain the frequency range containing the mu (μ: 8–12Hz) and beta (β: 12–30Hz) frequency bands. Both the mu (μ) and beta (β) bands are strongly associated with event-related desynchronization (ERD) during MI performance, indicating that they are particularly important for MI detection. Although the frequency range of the mu (μ) band overlaps with the conventional alpha (α) frequency range, their spatial distribution and neurological functionality should be distinguished. The mu (μ) band is specifically associated with sensorimotor cortical activity and is predominantly observed over the sensorimotor cortex, as indicated by electrode positions C3, C4, and Cz. In contrast, the alpha (α) band is most common over the occipital lobe and typically exhibits suppression during mental tasks. In the next step, a notch filter (50Hz) was integrated into the process to eliminate power-line interference from the European electrical grid. Real-time artifact-rejection mechanisms based on standard-deviation threshold (<$10^{-6}$) and signal-railing thresholds (>90%) were embedded into the code to detect EEG channels with insufficient signal variation or excessive clipping and to identify data segments potentially affected by acquisition-related artifacts. Data segments of 1 s were evaluated for this purpose.

### 2.4. Deep Learning Model Fitting and Classification

For the classification of the processed two-class MI data, a Convolutional Neural Network (CNN) architecture optimized for EEG signals—specifically, EEGNet [38]—was used and trained.

The EEGNet model was implemented without fundamental architectural modifications and configured to process the spatial-temporal structure of the specific 8-channel input matrix across the time-series segments within the scope of this case study. The final decision-making layers were fitted using the personalized training dataset of 1800 total trials. The model achieves an offline training accuracy of 89%. After the training, the model was stored within a JSON file for subsequent use. The trained model then operates as the central logic unit of the framework during the performance of the developed serious game and is embedded in the Python Backend system. Given the state of the gameplay, MI tasks must be executed by the user, recorded via the Cyton board, preprocessed, and then transmitted to the fitted EEGNet model for online estimation. The classification output is subsequently translated into the corresponding steering commands (Left vs. Right) for the Unity-based VR application, thereby establishing the game’s steering mechanism.

### 2.5. Immersive VR Serious Game Interface

The serious game, *Formula Mind*, was developed using the Unity game engine [41] (v6.2, Unity, San Francisco, CA, USA) and deployed as a standalone application on the Meta Quest 2 [37] headset (Menlo Park, CA, USA). The serious game simulates a racing game environment designed to support cognitive therapy for individuals with ADHD. The control loop combines two distinct mental modalities:

1. Directional Control (MI): Real-time steering commands, estimated by the backend’s EEGNet [38] architecture and based on recorded brain-signal traces from the participant’s scalp, are transmitted via network sockets to steer the vehicle left or right.
2. Velocity Control (Concentration Level): Simultaneously, the user’s focus is monitored via cognitive state metrics from the BrainFlow API [40]. Higher concentration levels translate to increased vehicle speed, enabling the user to directly influence the game duration. An optional visual progress bar (concentration-level color bar) visualizes the current speed.

### 2.6. Usage of Generative AI Tools

**Figure 1:**
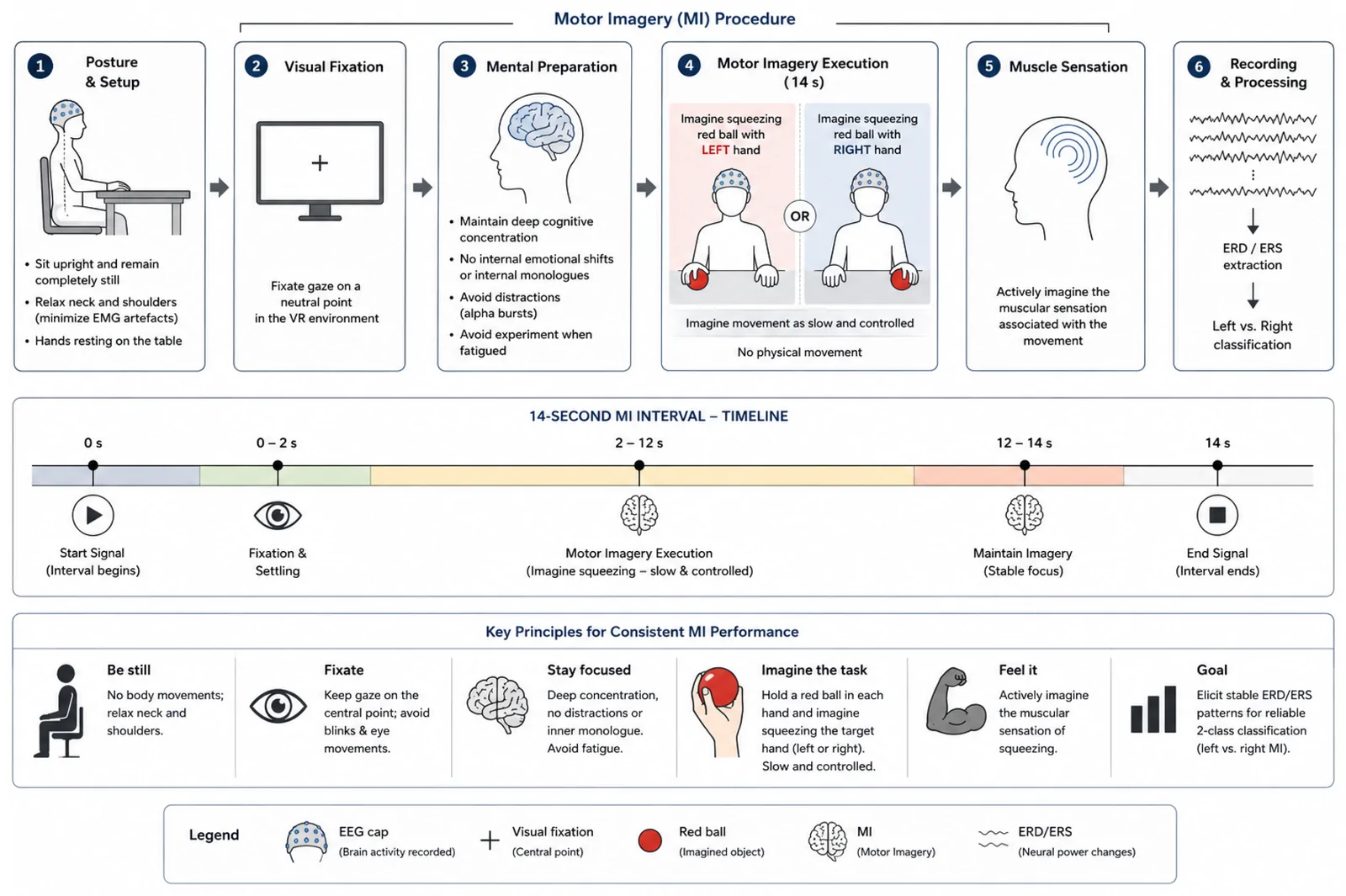
Visual representation of the Motor Imagery Process (generated with ChatGPT-5.5).

In accordance with the editorial guidelines for scientific publications, the authors declare the use of advanced Generative AI tools (specifically, the large language model Gemini developed by Google [42] and ChatGPT-5.5 by OpenAI [43]) during the preparation of this manuscript and the underlying work. The AI assistant was strictly utilized as a collaborative writing and formatting tool. In addition, it was used to create various representations to support processes and strengthen the overall understanding of this work (see Figure 1). Crucially, the generative AI was not used for data collection, experimental execution, literature interpretation, or the derivation of scientific conclusions. All empirical results, hardware configurations, classification accuracies, and architectural logic remain entirely the original work of the human researchers.

## 3. Results

### 3.1. System Architecture Overview

**Figure 2:**
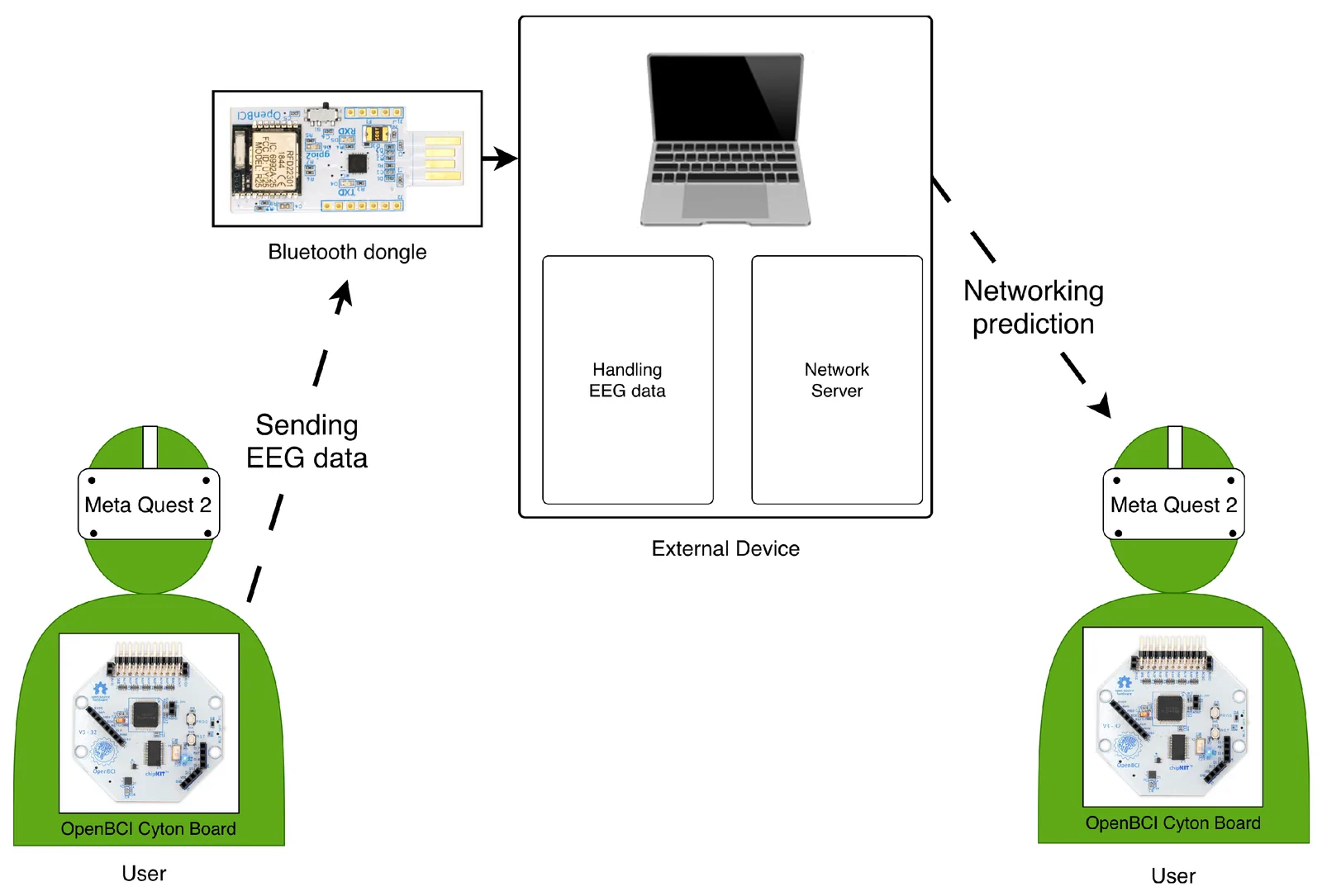
System architecture of the mind-driven VR serious game illustrating the data pipeline from 8-channel EEG acquisition to real-time classification.

The integrated BCI and VR framework consists of three primary interconnected components: (i) the Data Acquisition Hardware, (ii) the Python-based Backend System for signal processing and machine learning classification, and (iii) the Immersive VR Frontend Application. Communication between the standalone VR HMD and the processing backend is established over a low-latency network connection via local sockets, enabling real-time control without compromising the mobile architecture. Figure 2 represents an overview of the communication workflow.

### 3.2. Neurophysiological Rationale

The technical ecosystem engineered for this case study establishes a lightweight, highly responsive, and mobile BCI architecture optimized for real-time applications. The hardware consists of the OpenBCI Cyton board, an 8-channel neural interface equipped with a 32-bit processor and a 24-bit analog-to-digital converter (ADC) (Texas Instruments, Dallas, TX, USA).

The board captures relevant neurophysiological signals in the range of 10 to 100 µV and transmits them wirelessly via a proprietary, programmable Bluetooth Low Energy (BLE) USB dongle plugged into a computer to the running Python-based Backend system, programmed throughout this scope. By decoupling the heavy computational workload of signal acquisition, preprocessing, and classification from the standalone VR HMD and offloading it to a machine learning model, the system maintains a low-latency execution loop while preserving the user’s absolute physical mobility.

For a non-invasive BCI system operating with a sparse sensor configuration, the electrode placement is highly critical. MI relies on the activation of sensorimotor rhythms localized within the primary sensorimotor cortex, specifically characterized by power fluctuations in the mu (μ: 8–12Hz) and beta (β: 12–30Hz) frequency bands as well as the alpha (α: 8–12Hz) band. When a user imagines a kinesthetic movement, a lateralized suppression of amplitude occurs over the contralateral hemisphere, known as Event-Related Desynchronization (ERD). Therefore, to effectively capture this phenomenon while not neglecting the relevant frequency bands required for measuring concentration without the density of a 19-channel clinical cap, an optimized 8-channel layout based on the international 10–20 system was deployed. The selected electrode placements and their corresponding input pin associations are presented in Table 1.

The central channels (C3,C4,andCz) are the most essential nodes of this architecture, as they directly overlay the precentral gyrus and the primary sensorimotor cortex, which are most relevant to motor-related tasks. Channels C3 and C4 map the lateralized activation profiles required to differentiate left-hand from right-hand motor imagination. The frontal electrodes (F3,F4,andFz) were strategically integrated to monitor prefrontal cognitive activity, serve as structural anchors for artifact-rejection networks (such as ocular blink detection), and assess the user’s focus or concentration. Furthermore, these positions allow the capturing of beta (β) waves. Lastly, the occipital channels (O1andO2) provide a continuous baseline of visual alpha rhythms (8–12Hz), which are crucial for detecting baseline cognitive shifts and assessing general relaxation states. In contrast to the mu (μ: 8–12Hz) band, which overlaps in frequency range with alpha activity, alpha activity is predominantly observed over posterior visual cortical regions and reflects distinct cognitive and attentional states. Table 2 provides an overview of the most relevant frequency bands for MI classification, describing their associations and corresponding electrode positions according to the international 10–20 system.

### 3.3. Data Acquisition, Impedance Matching, and Preprocessing

**Figure 3:**
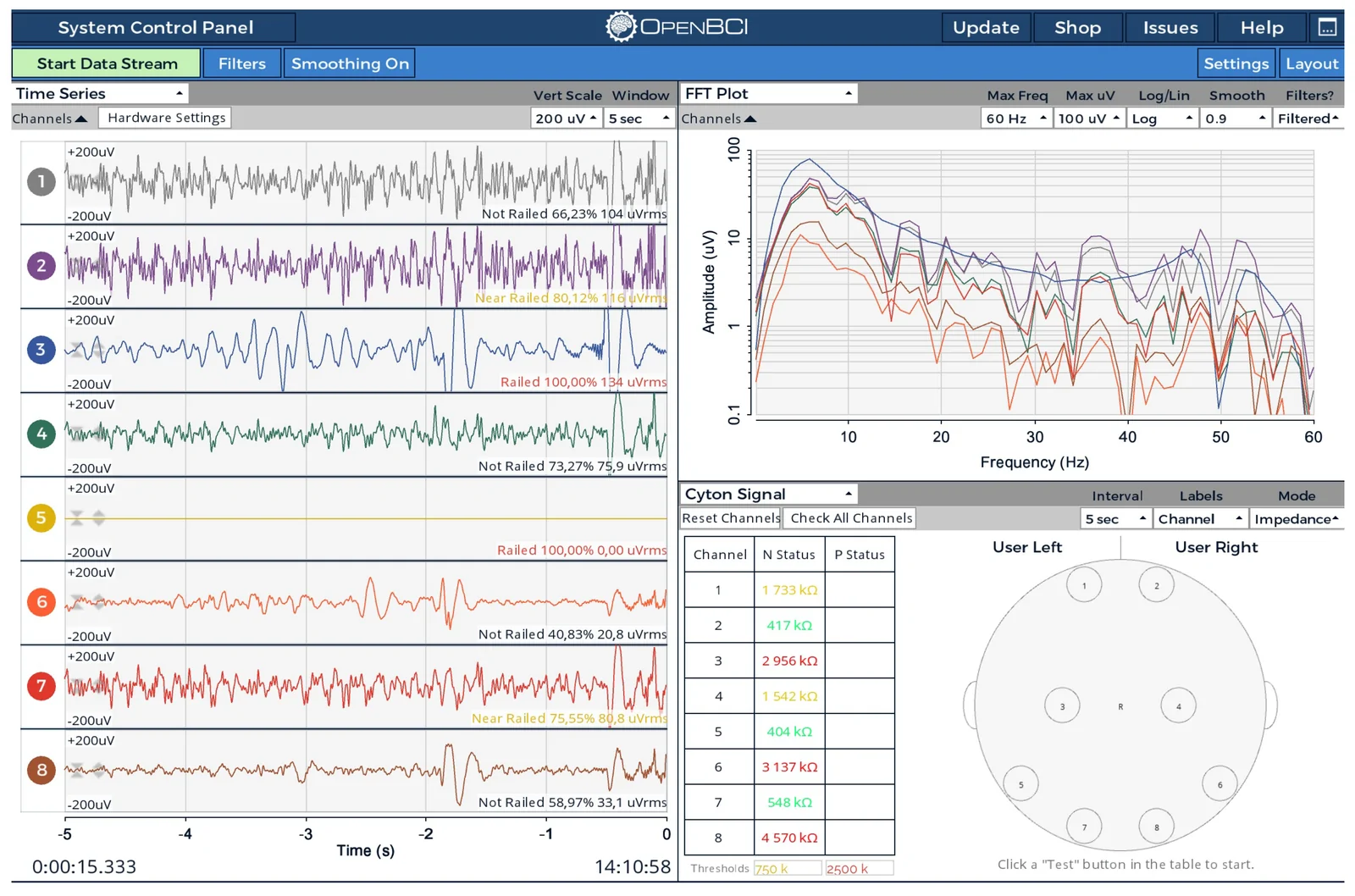
Recorded EEG information without the defined conditions.

To achieve a high signal-to-noise ratio (SNR) in a non-shielded laboratory environment, a rigorous data acquisition protocol was mandated. Prior to starting any runtime session, system initialization, hardware testing, and physical validation were executed using the official OpenBCI GUI. The software enabled real-time visualization of channel performance and calculation of skin–electrode contact quality via continuous impedance measurements. Maintaining individual electrode impedance values strictly below 750 kΩ was necessary to eliminate the risk of channel railing. Railing occurs when high impedance induces severe voltage offsets, causing the signal to reach the ADCs’ saturation boundaries (100% railing), resulting in irreversible signal clipping and the loss of informative EEG data. To further reduce the likelihood of clipping, the implemented “Gain” value of the board was set from 24 to 12 prior to each measurement session. The “Gain” value of the OpenBCI Cyton board sets the amplification factor applied to the input signal prior to analog-to-digital conversion. A moderate gain improves the measurability of weak biological signals, such as EEG, while keeping the amplified signal within the ADC’s required range. Using higher gains in the presence of substantial noise increases the likelihood that the amplified signal will clip. This phenomenon is visible in Figure 3 at channel 5.

**Figure 4:**
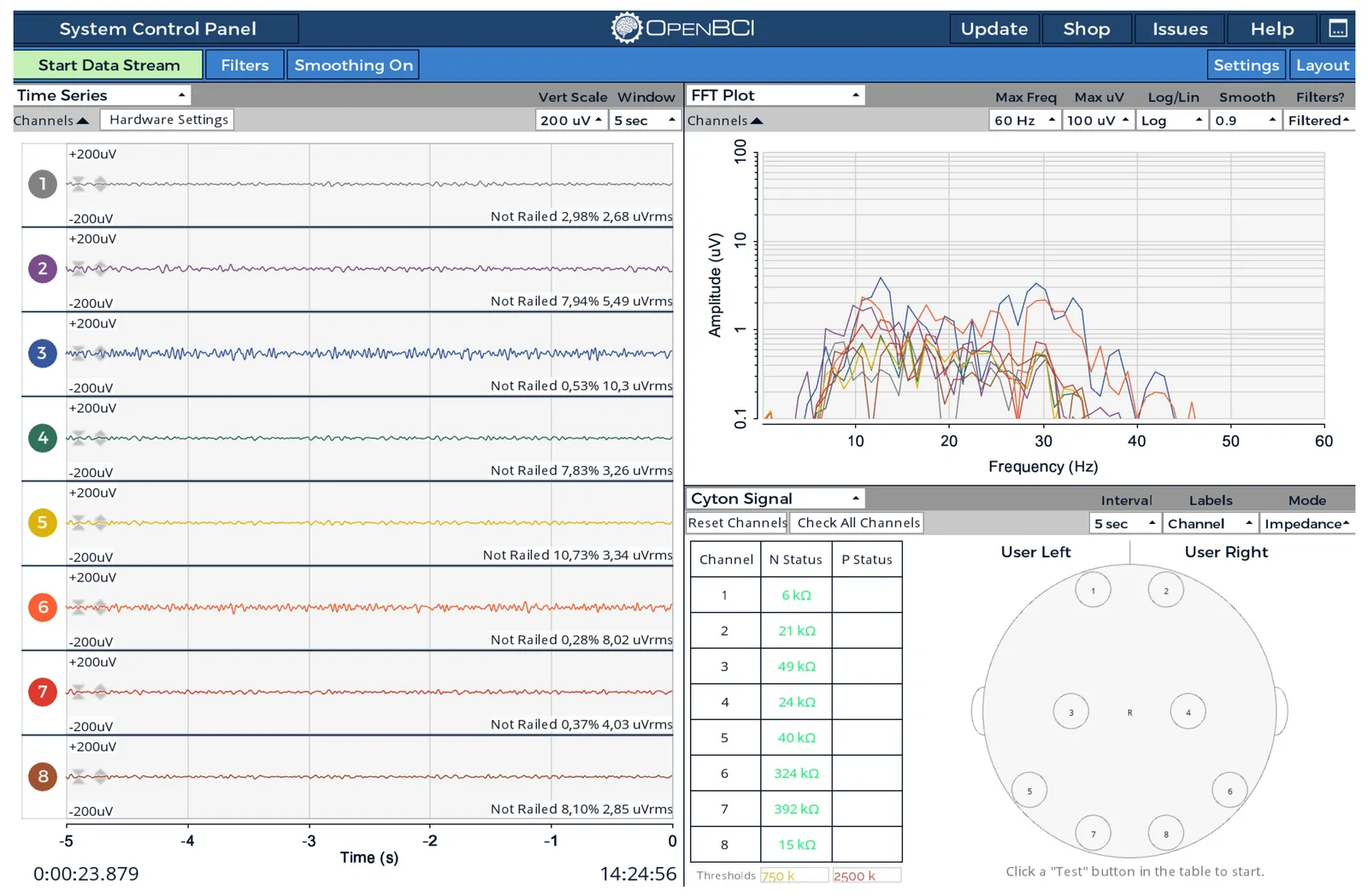
Recorded EEG information with the defined conditions.

The physical integration of the EEG cap underneath a Meta Quest 2 [37] VR HMD introduced severe mechanical challenges. The strap of the VR glasses applied localized mechanical tension to the underlying cables, thereby inducing extreme motion artifacts. To mitigate this limitation, conductive gel was first applied to the electrodes, which were fixed to the used EEG cap manufactured by BrainBit. To further enhance signal quality by reducing induced noise, spacers were placed between the cap and the HMD strap to avoid mechanical pressure on the cables connecting each EEG electrode to the selected OpenBCI Cyton board. Under these defined conditions, the signal quality was enhanced, represented in Figure 4.

Once physical signal validation was successfully completed in the GUI, streaming control was fully transferred to the Python Backend system via the open-source BrainFlow SDK. This programmatic architecture accesses the hardware data stream directly, allowing immediate processing of the received data. Using Python code, the internal board gain of the Cyton board’s programmable gain amplifiers was dynamically adjusted during connection establishment between the Backend system and the system, thereby optimizing resolution without inducing clipping. The continuous data stream, sampled at 250Hz, was passed through a digital preprocessing pipeline right before storing or handling the signals (4th-order Butterworth bandpass filter spanning from 8 Hz to 30 Hz; a notch filter at 50 Hz; and real-time artifact detection based on a 1 s data window using standard-deviation and signal-railing thresholds).

### 3.4. MI Presentation

For the MI task, the imagined movement consisted of a mental hand-closing movement (left vs. right). The duration of each continuous MI measurement interval was fixed at 14 s and tracked by the implemented system. This window length was chosen to capture the full temporal evolution of the ERD/ERS patterns, allowing the neural networks to evaluate stable power shifts over a prolonged mental execution block. In order to ensure a consistent execution of the MI task across the individual measurements, a protocol was established, and a precise procedure for the mental imagination of the defined movements was specified as follows:

- Users were instructed to remain completely still, sitting upright with relaxed neck and shoulder muscles to minimize high-frequency electromyography (EMG) artifacts.
- Fixation of the gaze on a neutral point was mandatory to suppress ocular artifacts caused by excessive eye blinks or movements.
- A consistent level of cognitive concentration had to be maintained without sudden distractions. Furthermore, participants were instructed to avoid performing the experiment when fatigued.
- The participant was instructed to place both hands on the table and imagine holding a red, soft ball in each hand. Depending on the measurement condition (left vs. right M), the participant then had to imagine squeezing the ball with the corresponding hand. Particular attention had to be paid to imagining the movement as slow and controlled.
- Instructions were given to actively imagine the muscular sensation associated with the individual movements during the MI performance.

The overall process is shown in Figure 1.

### 3.5. Training Dataset Generation and Environmental Control

The training phase focused on establishing a balanced, personalized dataset to capture individual neural patterns during MI performance, ensuring a sufficient dataset for developing reliable classification models. During data acquisition, the implemented software pipeline continuously recorded the EEG stream, while time-based numerical markers were automatically inserted into the data stream via the backend architecture whenever the corresponding MI measurement activation button was triggered via the user interface. These markers precisely indicated the beginning and end of each MI trial, thereby enabling an accurate temporal segmentation of the continuous EEG recordings into class-labeled MI epochs. To provide a sufficiently large subject-specific dataset to support machine-learning and deep-learning training, an extensive data-collection protocol was implemented. The final compiled training dataset comprised a total of 900 recorded training trials per class, resulting in 1800 fully labeled 14 s 8-channel matrices.

To eliminate potential anticipation and habituation effects, such as reduced cognitive and neural adaptations or structural habituation, the order of the left and right MI trials was pseudo-randomized throughout the measurement procedure. This strict arbitrariness ensured that the classifier was fitted purely on the sensory-motor variations rather than temporal pattern prediction. Furthermore, all training data acquisition trials were performed at the same location and under nearly identical environmental conditions, avoiding the activation of electronic devices that could easily generate electromagnetic interference with the measured EEG signals.

### 3.6. Feature Extraction and Offline Classification

Prior to training the machine learning models, such as Support Vector Machine (SVM) or Linear Discriminant Analysis (LDA), informative features were extracted from the recorded EEG signals. Therefore, feature extraction was systematically evaluated in both the frequency and spatial domains. Power Spectral Density (PSD) estimation using Welch’s method was applied to compute the logarithmic power distribution in the 8–30Hz frequency band for each recorded EEG channel. In addition, Filter Bank Common Spatial Patterns (FB-CSP) were applied to exploit the spatial configuration of the 8-channel EEG layout and to calculate optimal spatial filters that maximize the variance differences between the left- and right-handed MI classes, thereby enhancing the discriminative spatial patterns across the recorded EEG trials.

For the final classification phase, three classification architectures were implemented and evaluated using the same subject-specific training dataset: Linear Discriminant Analysis (LDA), Support Vector Machines (SVM) utilizing a radial basis function (RBF) kernel, and EEGNet [38], a specifically designed form of the Convolutional Neural Network (CNN) tailored for raw EEG data classification. The offline classification results for each model were evaluated for accuracy. The obtained results are presented in Table 3.

The conventional machine learning models, LDA and SVM, achieved offline classification accuracies between 65% and 68%, respectively, depending on the feature extraction method used. This limitation is attributed to their inability to resolve complex dependencies across the temporal and spatial characteristics of an EEG signal recorded with an 8-channel electrode system. The fitted EEGNet [38] architecture achieved the highest classification accuracy of 89% for the primary subject using the personalized training dataset. By applying depthwise and separable convolutions directly to the filtered EEG time series, EEGNet [38] learned discriminative spatio-temporal representations without requiring an explicit feature-extraction stage.

### 3.7. Real-Time Validation

To evaluate the proposed system under real-time conditions, the VR serious game “Formula Mind”, developed throughout the scope of this paper, was employed as the evaluation platform. The game was designed to explore a potential neurofeedback-based therapeutic application for individuals struggling with ADHD. During gameplay, the system requested MI measurements to steer the avatar (a virtual racing car) to the left or right. These measurements were recorded by the BCI device and subsequently translated to the trained EEGNet model, which achieved the best offline classification accuracy. The recorded MI sequences were then classified by the model in real-time. The results were converted into digital commands and transmitted back to the serious game, enabling the virtual car to be controlled exclusively through MI and the participant’s level of cognitive concentration.

The online evaluation was conducted with five voluntary participants (listed in Table 4), each of whom completed at least two full gameplay sessions and made at least 19 valid MI classifications. Furthermore, the evaluation was also performed by the primary subject whose EEG recordings had previously been used to train the classification model. The evaluation results of the online classification are presented in Table 4.

Owing to inter-subject variability in brain anatomy and individual neural activity patterns, the five independent participants achieved an overall classification accuracy of up to 50%. In contrast, the primary subject, whose data were exclusively used to train the EEGNet model, confirmed the offline performance observed during the training phase of the EEGNet model and achieved an online classification accuracy of 90%. Initial attempts to apply transfer learning by freezing the pre-trained EEGNet layers and subsequently fine-tuning the prediction layer using 100 training trials per class for participants were not successful and failed to achieve the desired classification performance.

Nevertheless, the evaluation results from the training subject demonstrate that reliable MI classification can be achieved with a low-cost, 8-channel BCI system while maintaining high classification accuracy. However, due to the inherent susceptibility of EEG signals to noise, as well as inter-subject differences in brain anatomy and functional neural activity that also depend on the participant’s current mental state, successful MI classification requires a sufficiently large and representative subject-specific training dataset to learn EEG patterns. Furthermore, if the model is intended to be applied across multiple participants, the training dataset must provide sufficient inter-subject variability to address neural differences.

## 4. Discussion

The primary objective of this case study was to evaluate the operational feasibility of a non-invasive BCI coupled with a standalone VR serious game (Formula Mind) for cognitive therapy. The empirical results of the case study confirm that an 8-channel consumer-grade configuration (F3,F4,C3,C4,Cz,Fz,O1,O2) provides sufficient signal information to capture sensorimotor frequencies for a 2-class MI task. The implemented EEGNet model demonstrated the ability to learn complex spatio-temporal brain pattern representations from recorded EEG time signals, achieving higher classification accuracy than the conventional machine learning models LDA and SVM. The online classification accuracy result of 90%, achieved by the primary subject, demonstrates that reliable real-time MI classification can be achieved using subject-specific training data and a low-cost BCI system without requiring high-end clinical hardware for real-time measurement. However, the substantially lower accuracies observed for the five additional participants indicate that this performance was subject-specific and cannot be generalized to unseen users based on the dataset used.

Furthermore, the suggested dual-modality control concept, which combines two distinct mental-state estimates, demonstrated the feasibility of simultaneously integrating multiple cognitive modalities within a VR-based BCI application. Compared to existing serious game approaches, which typically rely on a single signal control modality or muscle-based artifacts, the proposed system combines MI for directional steering control with continuous concentration estimation for vehicle speed adjustment, enabling the simultaneous use of two independent cognitive control modalities within a single VR serious game.

### 4.1. Technical and Physiological Challenges

During the developmental phase, a limitation of the overall system regarding EEG data acquisition and its visualization becomes apparent. Although the proprietary OpenBCI GUI [36] was suitable for the initial hardware configuration, verification of electrode impedance, as well as visual inspection of the recorded EEG signals, it is not designed for real-time applications such as a VR serious game, where instantaneous feedback and fully closed-loop control are mandatory. Therefore, implementing a Python-driven Backend system was required to directly acquire, process, mark, and stream the EEG data in real time.

Consequently, the EEG data acquisition technology was completely migrated to a code-based implementation using the BrainFlow SDK [40] in Python. This approach enabled the EEG data to be directly preprocessed and marked, and then stored in the corresponding memory MI-class directory. However, replacing the GUI through the Backend system within the scope not only eliminated the integrated visual feedback regarding signal quality and electrode failures, leading to the necessity of a custom-implemented user interface, visualization technology of the signal, and a continuous signal quality check, but also raised another major disadvantage at the stage of correct signal acquisition.

While the OpenBCI GUI [36] automatically applies preprocessing to the recorded data before visualizing it, the BrainFlow SDK [40] only records raw and unfiltered data containing large DC offset and low-latency noise, leading to the acquisition of values exceeding 100,000 in the unit of Volts, respectively µV, which seems quite implausible. Consequently, a dedicated preprocessing pipeline consisting of band-pass and notch filtering was implemented to transform the received signals into the expected range of 1–2 µV to 100 µV.

Despite the transient filter response, elevated signal amplitudes were still observed, especially at the start of each acquired data stream. To prevent false-positive detections from the implemented signal-quality check, the first few seconds of each acquired data stream were discarded during preprocessing. This resulted in a stable, primarily artifact-reduced EEG signal suitable for subsequent storage and visualization as well as feature extraction and classification.

Within the scope of this work, a strong dependence between EEG signal quality and the participant’s mental state was observed. In particular, obtaining EEG recordings of sufficient signal quality was considerably more challenging during evening sessions than during measurements after a restful sleep, which primarily yielded satisfactory signal quality. These observations are attributed to the participant’s mental state and level of fatigue, representing the strong influence of psychological stability and cognitive concentration on neural activity. Therefore, the quality of informative EEG signals may vary substantially within the same participant, even over the course of a single day.

Similar phenomena were observed during the individual training sessions. While the signal quality at the beginning of each session was consistently high and all signal-quality checks were successfully passed, the number of discarded recordings increased noticeably after continuous measurements exceeding one to two hours. Furthermore, both training and online evaluation sessions longer than one hour were characterized by significantly longer preparation times to achieve satisfactory signal quality and by a higher number of incorrectly classified MI predictions, further indicating that mental states considerably influence the ability to measure informative EEG signals.

Acquiring sufficient EEG signal quality requires careful hardware calibration. Due to high input impedance between the electrodes and the scalp, the recorded signals frequently exceeded the saturation boundaries of the analog-to-digital converter (ADC), leading to 100% channel railing and signal clipping. To address this behavior, the OpenBCI Cyton board includes a programmable gain amplifier (PGA), which amplifies the analog input signal before it is converted to digital. This amplification improves the signal-to-noise ratio and enables low-amplitude EEG signals in the µV range to dominate the signal and be presented with high resolution. Furthermore, an appropriate gain factor setting avoids signal clipping from head or body movements while preserving sufficient sensitivity to capture low-amplitude EEG activity, making the correct selection of a gain factor an important step in accurate brain wave measurements. For the proposed system, a PGA gain setting of 12 was selected, as it provided a suitable balance between preventing signal clipping and preserving sufficient measurement resolution.

Another practical challenge arose from loose electrode cables and the mechanical interaction between the EEG cap and the selected HMD. By tightening the headset strap, mechanical pressure was exerted on both the underlying cables and the electrodes, occasionally causing individual electrodes to shift and introducing additional artifacts into the recorded EEG signals. Additionally, loose cables connecting the electrodes to the board generated artifacts during head and body movements due to mechanical displacement and electromagnetic interference. Therefore, to ensure a stable measurement setup, all electrode cables were carefully routed and secured to the EEG cap using elastic bands, while avoiding unnecessary cable overlap. Applying conductive gel to the electrodes improved contact between the electrodes and the scalp and reduced impedance. Finally, spacers were placed between the VR HMD strap and the EEG cap to reduce mechanical tension on the electrode cables, thereby helping maintain stable signal quality even when an HMD is worn above the EEG cap. No further issues attributable to the VR HMD, such as interference from its integrated Wi-Fi/Bluetooth module or from operating the device itself, could be detected. By applying conductive gel, using the necessary spacers, and adjusting and securing the loose cables, satisfactory signal quality was achieved under the stated conditions, provided that older electronic devices including refrigerators or air-conditioning units, which are potential sources of electromagnetic interference, were not operating nearby.

### 4.2. Limitations and Future Directions

A fundamental limitation of this case study is its subject-specific functionality. Individual differences in brain structures and functional neural activity lead to considerable variation in recorded EEG signals, limiting the direct transferability of trained classification models across participants. This behavior was also observed during the evaluation phase of the online classification with five participants. A subsequent transfer learning experiment also failed, demonstrating that an EEGNet model trained on the complete training dataset of a single subject requires substantially more than 100 MI trials per class when applied to a new participant and the feature extraction layers are frozen, while only retraining the final classification layer. Under these conditions, the classification accuracy of the pre-trained EEGNet model decreased to 57%, indicating that the proposed model requires a sufficiently large and representative training dataset to reliably capture individual EEG patterns. Furthermore, achieving reliable cross-subject classification will require greater inter-subject variability in the training data to improve the classification model’s generalization capability.

Another limitation of the proposed work is the requirement for conductive gel and the permanent fixation of the electrode cables on the EEG cap. Since the conductive gel must be applied to the electrodes before the EEG cap is placed on the participant’s head, some of the gel is unintentionally displaced into the hair during cap placement, reducing the intended effect of lowering electrode impedance. Furthermore, the conductive gel leaves visible residues on the scalp, hair, and the EEG cap, necessitating cleaning after each measurement session. Because rerouting the cables while still achieving satisfactory signal quality afterwards is quite challenging, permanently fixing the cables to the EEG cap makes cleaning or disinfecting the cap difficult. Consequently, maintaining the EEG cap becomes more difficult, resulting in hygiene restrictions and limiting the practical evaluation of the proposed system with a larger number of participants.

These mechanical restrictions, particularly the use of conductive gel and the required cable routing, result in a time-consuming preparation process and limit the usability of the proposed system. Future work should therefore investigate alternative hardware configurations, including dry electrodes and improved cable-management solutions, especially before evaluating the system with children or individuals with ADHD. Reducing setup time and complexity may help prevent impatience, fatigue, and reduced compliance during preparation.

Within the scope of this case study, no quantitative end-to-end latency measurement was performed. The overall latency depends on several setup-specific components and their communication paths, including the Bluetooth connection between the Cyton board and the Python-based Backend, as well as the Wi-Fi connection between the VR HMD and the Backend. Nevertheless, latency is a crucial factor for providing responsive real-time neurofeedback. Future work should therefore measure the end-to-end latency of the complete processing pipeline, from the acquisition of EEG data during MI or concentration tasks to the delivery of the estimated value and corresponding feedback in the VR application. Although no perceptible delay was observed during the evaluation phase, this qualitative observation does not replace a systematic quantitative latency assessment and should therefore be a focus of future work.

## 5. Conclusions

This case study presented the design, implementation, and technical evaluation of a non-invasive, mind-driven VR serious game designed as a therapeutic intervention for individuals with ADHD. The proposed approach demonstrates that reliable EEG acquisition and preprocessing can be achieved using a low-cost, consumer-grade 8-channel EEG setup based on the OpenBCI Cyton board. Using 900 MI training trials per class, acquired from a single primary subject, the implemented EEGNet model achieved an offline left/right classification accuracy of 89% for the same subject. The subsequent online evaluation within the developed VR serious game “FormulaMind” confirmed these results, achieving an online classification accuracy of 90% for the primary subject. Through this, the case study demonstrated that the investigated configuration can support subject-specific real-time MI classification under controlled conditions.

Furthermore, integrating continuous concentration estimation into the gameplay enabled the simultaneous use of two cognitive control modalities within a single application. This demonstrates the feasibility of multimodal BCI interaction in an immersive VR environment using a low-cost, consumer-friendly 8-channel EEG system. Additionally, discussing the practical challenges encountered throughout this work and presenting possible solutions contributes to the existing literature by providing insights for future research and practical implementations of comparable BCI systems.

Despite the classification performance achieved, significant challenges regarding model generalization and practical setup overhead remain. Both the online evaluation with 5 additional healthy participants and the subsequent transfer learning experiment demonstrated that the trained classification model cannot be directly transferred between users, especially when it is trained on data from a single subject. The reduction in classification accuracy to 57% during the transfer learning experiment confirmed this, indicating that brain signals are highly subject-specific. Therefore, reliable MI classification requires sufficiently large and subject-specific training datasets for each participant that capture the unique neuroanatomical structures and neural functional activities of each individual brain. Furthermore, mechanical tension between the VR headset strap and the electrode cables, high skin–electrode impedance, and environmental electromagnetic noise from nearby electronic devices highlight the main problems in acquiring brain signals, considerably influencing the quality of the recorded EEG and distorting the informative data. Consequently, a conductive gel to reduce electrode impedance was required, which raised another problem: hygiene restrictions and complex preparation routines for the current suggested system, thereby limiting spontaneous deployment.

Future work should therefore focus on improving the generalized capability of the classification model by collecting larger cross-subject training datasets and investigating transfer learning strategies for subject-independent MI classification. Furthermore, alternative hardware configurations, including dry electrodes and optimized cable management, should be tested and evaluated to mitigate existing hygiene restrictions, thereby enabling testing of the system with a larger group of participants and reducing preparation time.

## Figures and Tables

**Table 1:** Electrode Placements and Functional Assignment of the 8-Channel BCI.

Input Pin	Electrode Placement	Reference Signal	For Motor Imagery	For Concentration Level
SRB	A1/A2	Yes	No	No
N1P	F4	No	Yes	Yes
N2P	C4	No	Yes	No
N3P	O2	No	Yes	Yes
N4P	Fz	No	Yes	Yes
N5P	Cz	No	Yes	No
N6P	O1	No	Yes	Yes
N7P	C3	No	Yes	No
N8P	F3	No	Yes	Yes
Bias	A1/A2	Yes	No	No

**Table 2:** Representation of the most relevant frequency bands.

Band	Frequency	Cortical Lobe	Electrode Placement	Representing
Alpha (α)	8–12 Hz (sinusoidal)	Occipital lobe	O1, O2	Mental tasks (↓)
Mu (μ)	8–12 Hz (non-sinusoidal)	Primary sensorimotor cortex	C3, Cz, C4	MI or processing (ERD ↓), No primary sensorimotor cortex activation (ERS ↑)
Beta (β)	12–30 Hz	Frontal & parietal lobe	Fp1, Fp2 F7, F3, Fz, F4, F8, P3, Pz, P4	MI or processing (ERD ↓), No primary sensorimotor cortex activation (ERS ↑)

**Table 3:** Cross-validated classification accuracies for 2-class MI using an 8-channel BCI device.

Model ID	Classification Model	Feature Extraction Method	Accuracy
M1	Linear Discriminant Analysis (LDA)	PSD (Welch)	65%
M2	Linear Discriminant Analysis (LDA)	FBCSP	68%
M3	Support Vector Machines (SVM)	PSD (Welch)	65%
M4	Support Vector Machines (SVM)	FBCSP	66%
M5	EEGNet (Deep CNN) [38]	Raw EEG data Matrix	89%

**Table 4:** Demographics and evaluation results of MI classification for all participants.

Participant	Age	Gender	Location	Number of Valid Classifications	Achieved Accuracy
PT1	25	Female	A	19	32%
PT2	52	Female	A	38	42%
PT3	25	Male	A	46	50%
PT4	57	Male	B	63	44%
PT5	25	Male	C	19	44%
PS	26	Male	A, B, C	123	90%

## Data Availability

Data available on request due to privacy restrictions. The raw data presented in this case study are available on request from the corresponding author. The data are not publicly available due to follow-up research and privacy of the participants.

## Abbreviations

The following abbreviations are used in this manuscript:

MI	Motor Imagery
ADHD	Attention-deficit/hyperactivity disorder
WHO	World Health Organization
BCI	Brain–Computer Interfaces
ALS	Amyotrophic Lateral Sclerosis
VR	Virtual Reality
EEG	Electroencephalography
HMD	Head-mounted displays
CNN	Convolutional Neural Network
ADC	Analog-to-digital converter
BLE	Bluetooth Low Energy
ERD	Event-Related Desynchronization
EMG	Electromyography
SCM	Support Vector Machine
LDA	Linear Discriminant Analysis
PSD	Power Spectral Density
RBF	Radial basis function
